# Supplementary material for: The Effect of Combined Atmospheric Plasma/UV Treatments on Improving the Durability of Flame Retardants Applied to Cotton
Source: Molecules. 2022 Dec 9;27(24):8737. doi: 10.3390/molecules27248737 (PMC9783300; doi:10.3390/molecules27248737)
Supplement: Supplementary file 1 [file molecules-27-08737-s001.zip › molecules-2072790-supplementary.pdf]

Supplementary Files

The effect of combined atmospheric plasma/UV treatments on improving the durability of flame retardants applied to cotton

M.Ayesh, A.R.Horrocks, B.Kandola; School of Engineering, University of Bolton, Bolton, UK, BL1 5AB

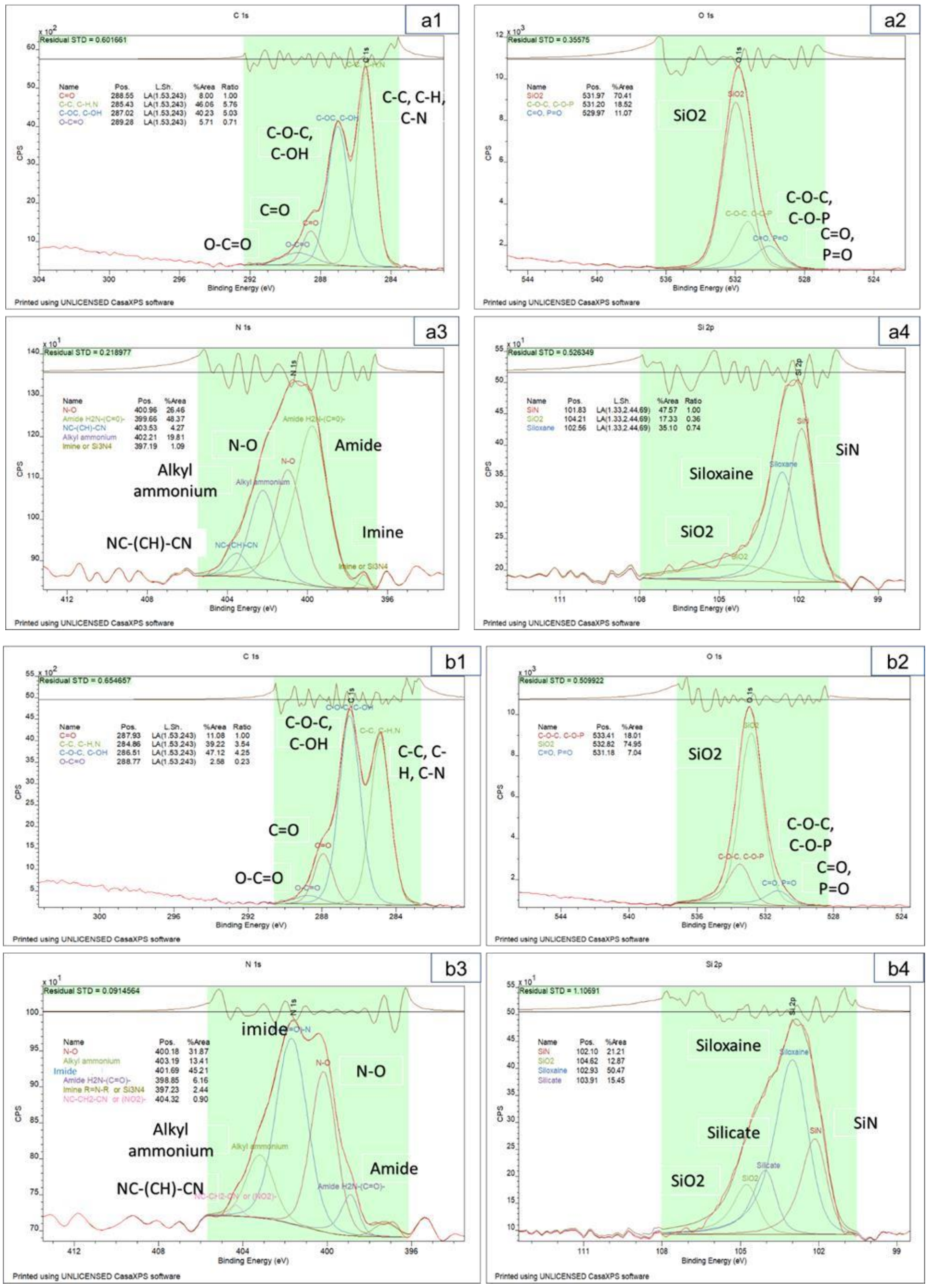

Figure S1: XPS high resolution spectra of the plasma/UV-treated cotton/FR fabric and Cot/FR after water-soaking. C1s, O1s, N1s and Si2p, respectively: (a1), (a2), (a3), (a4) Cot/DAP-urea-APTS-(ws), (b1), (b2), (b3), (b4) Cot/DAP-urea-APTS_PL(N_2 /O_2)-(ws)

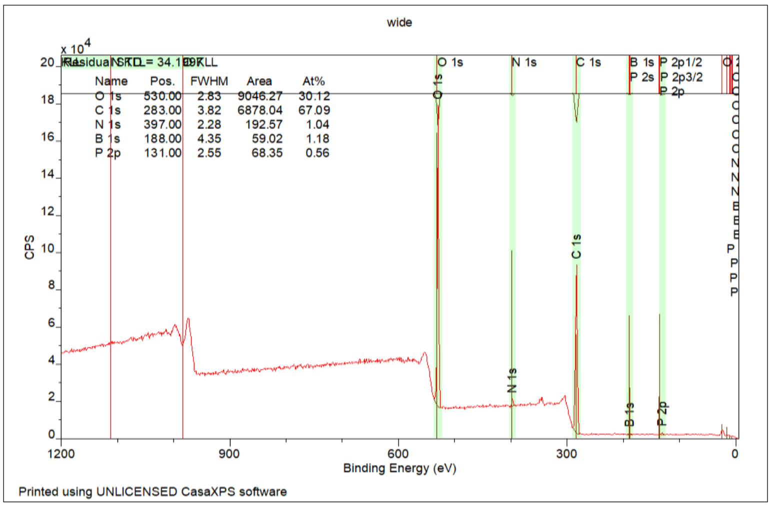

(a)

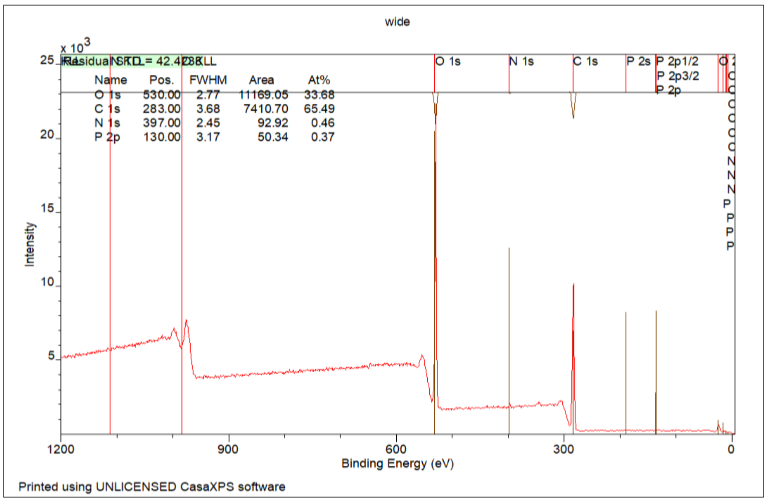

(b)

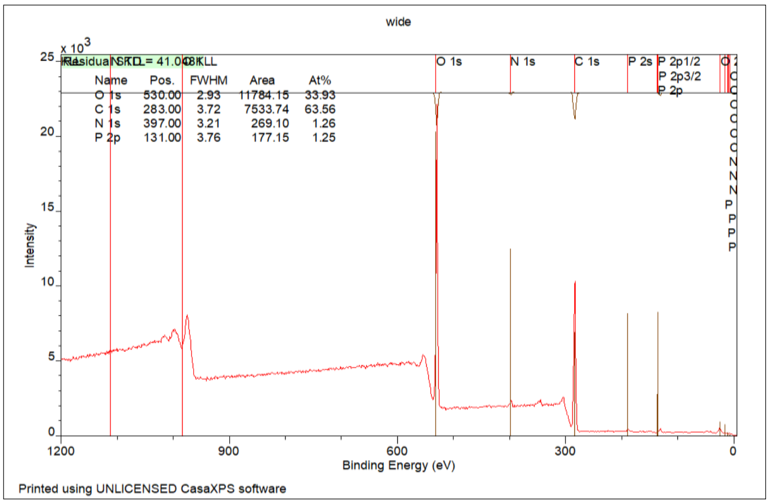

(c)

Figure S2: XPS spectra for Cotton/DBAP samples after water-soaking with and without plasma/UV laser treatment, (a) Cot/DBAP-(ws), (b) Cot_PL(Ar/CO_2)_DBAP_PL(N_2/O_2)-(ws) and (c) Cot/DBAP_ PL(N_2/CO_2)-(ws).

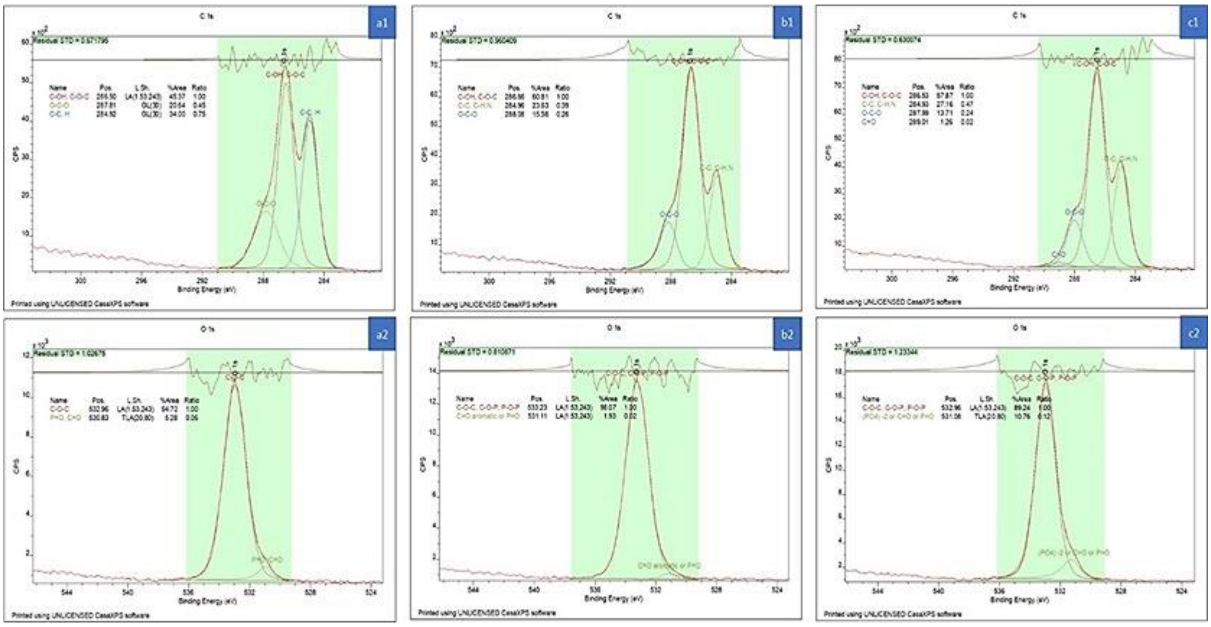

Figure S3: XPS high resolution spectra of the plasma/UV-treated cotton/FR fabric and control. C1s and O1s, respectively: (a1), (a2) Cot/DBAP, (b1), (b2) Cot_PL(Ar/CO_2)_DBAP_PL(N_2/O_2), (c1), (c2) Cot/DBAP_PL(N_2/CO_2)

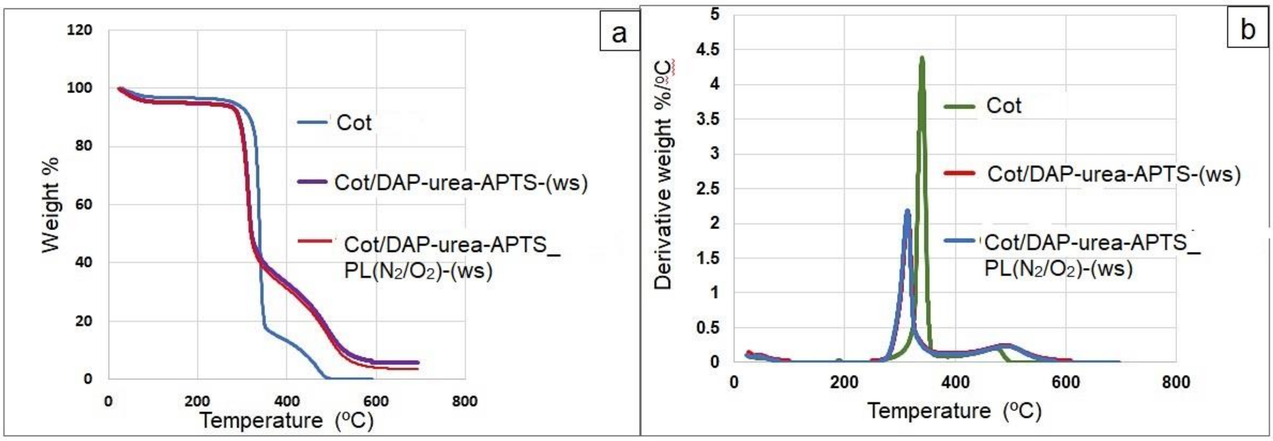

Figure S4: Thermal analysis traces of Cot/DAP-urea-APTS-(ws), Cot/DAP-urea-APTS_PL(N_2/O_2)-(ws) samples and cotton controls, after water-soaking. (a) TGA and (b) DTG; note that the DTG responses of Cot/DAP-urea-APTS-(ws) and Cot/DAP-urea-APTS_PL(N_2/O_2)-(ws) are almost superimposed (see also Table 8).

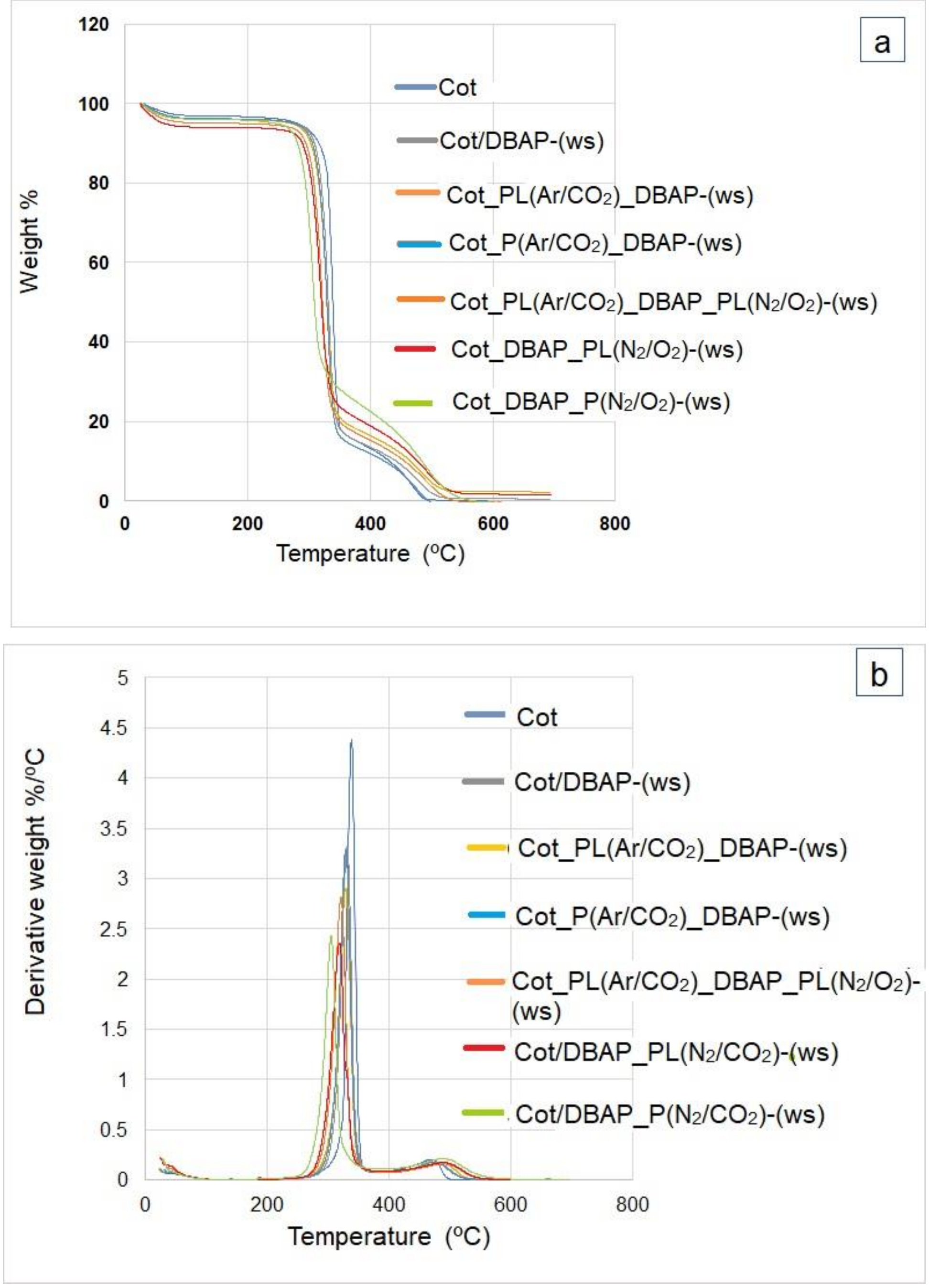

Figure S5: TA analysis for cotton (Cot) and Cot/DBAP samples before and after plasma/UV treatment after water-soaking.(a) TGA, (b) DTG (see also Table 8).

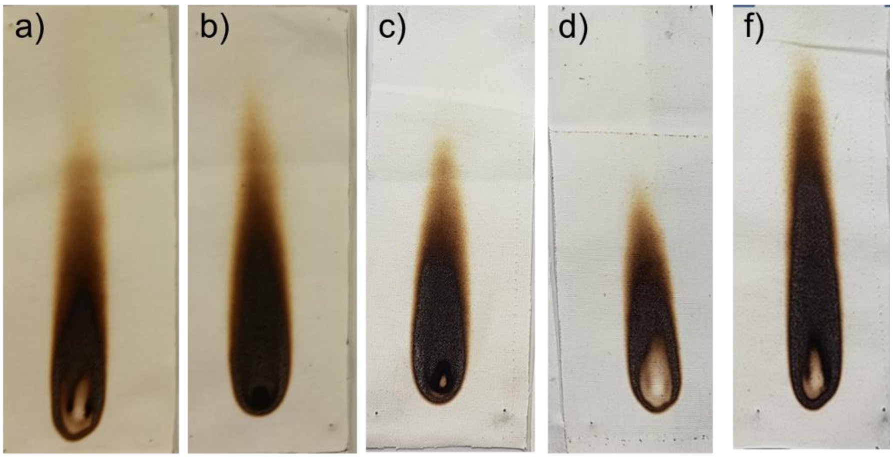

Figure S6: Vertical strip flammability test results before water-soaking for: (a) Cot/DBAP, (b) Cot_PL(Ar/CO_2)_DBAP, (c) Cot_P(Ar/CO_2)_DBAP (no UV laser), d) Cot/DBAP_PL(N_2/CO_2), (f) Cot_PL(Ar/CO_2)_DBAP_PL(N_2 /O_2)

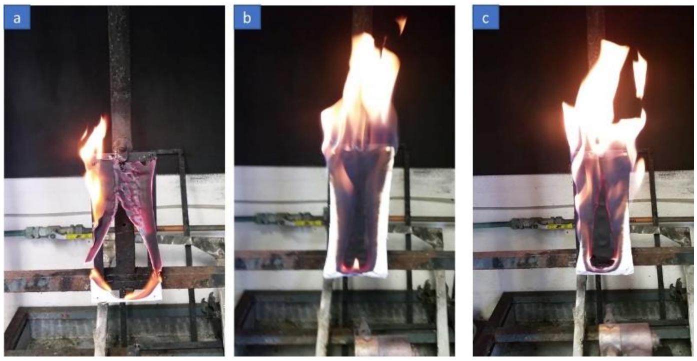

Figure S7. Vertical strip testing images after water-soaking selected samples after 10s ignition and a further 30s: (a) Cot, (b) Cot/DBAP, (c) Cot_PL(Ar/CO_2)_DBAP
